# Differential diagnosis of mild cognitive impairment of Alzheimer’s disease by Simoa p-tau181 measurements with matching plasma and CSF

**DOI:** 10.3389/fnmol.2023.1288930

**Published:** 2024-01-08

**Authors:** Ling Wu, Stephanie Arvai, Shih-Hsiu J. Wang, Andy J. Liu, Bin Xu

**Affiliations:** ^1^Biomanufacturing Research Institute and Technology Enterprise (BRITE), North Carolina Central University, Durham, NC, United States; ^2^Duke-UNC Alzheimer’s Disease Research Center, Durham, NC, United States; ^3^Department of Neurology, Duke University Medical Center, Durham, NC, United States; ^4^Department of Pathology, Duke University Medical Center, Durham, NC, United States; ^5^Department of Pharmaceutical Sciences, North Carolina Central University, Durham, NC, United States

**Keywords:** Alzheimer’s disease, mild cognitive impairment, p-tau181 biomarker, early diagnosis, matching plasma and CSF pre-mortem samples

## Abstract

Alzheimer’s disease (AD) is characterized by a long preclinical phase. Although late-stage AD/dementia may be robustly differentiated from cognitively normal individuals by means of a clinical evaluation, PET imaging, and established biofluid biomarkers, disease differentiation between cognitively normal and various subtypes of mild cognitive impairment (MCI) remains a challenging task. Differential biomarkers for early-stage AD diagnosis with accessible biofluid samples are urgently needed. Misfolded phosphorylated tau aggregates (p-tau) are present in multiple neurodegenerative diseases known as “tauopathies”, with the most common being AD. P-tau181 is a well-established p-tau biomarker to differentiate AD dementia from non-AD pathology. However, it is unclear if p-tau181 is capable of diagnosing MCI, an early AD stage, from cognitively normal subjects, or if it can discriminate MCI subtypes amnestic MCI (aMCI) from non-amnestic MCI (naMCI). Here we evaluated the capability of p-tau181 in diagnosing MCI from cognitively normal subjects and discriminating aMCI from naMCI subtypes. We collected matching plasma and CSF samples of a clinically diagnosed cohort of 35 cognitively normal, 34 aMCI, 17 naMCI, and 31 AD dementia cases (total 117 participants) with supplemental CSF Aβ42 and total tau AD biomarker levels and performed Simoa p-tau181 assays. The diagnostic capabilities of Simoa p-tau181 assays to differentiate these cohorts were evaluated. We found (i) p-tau181 can robustly differentiate MCI or aMCI from cognitively normal cohorts with matching plasma and CSF samples, but such differentiation is weaker in diagnosing naMCI from cognitively normal groups, (ii) p-tau181 is not capable of differentiating aMCI from naMCI cohorts, and (iii) either factor of Aβ or total tau burden markedly improved differentiation power to diagnose aMCI from cognitively normal group. Plasma and CSF p-tau181 levels may serve as a promising biomarker for diagnosing aMCI from normal controls in the preclinical phase. But more robust new biomarkers are needed to differentiate naMCI from cognitively normal cases or to discriminate between MCI subtypes, aMCI from naMCI.

## Introduction

Accumulation of tau neurofibrillary tangles (NFTs) and Aβ amyloid plaques are two neuropathological features of Alzheimer’s disease (AD) (Braak and Braak, 1991; Lee et al., 2001; Selkoe, 2001). NFTs are made from hyperphosphorylated tau, a neuron-enriched, microtubule (MT) associated protein. Under non-pathological conditions, tau is minimally phosphorylated, highly soluble and shows little tendency to form aggregates. After phosphorylation and other post-translational modifications (Wesseling et al., 2020), however, tau aggregates into insoluble, paired helical and straight filaments, tightly packed bundles of which constitute the NFTs thought to be neurotoxic and important in the pathogenesis of AD and related tauopathies. AD is a slowly progressing disease. It can take 15–20 years to develop noticeable neuropathological changes that lead to cognitive impairment and behavioral abnormalities (Vermunt et al., 2019). Therefore, early detection of AD is critical for devising novel treatment strategies that may prevent or slow down progression to severe dementia, which significantly impacts the quality of life and cost of care.

Mild cognitive impairment (MCI) is considered an early disease stage of AD where subtle cognitive changes can be detected by neurocognitive tests but the ability to perform daily activities independently is preserved. MCI is classified as amnestic MCI (aMCI) and non-amnestic MCI (naMCI), with the former primarily affecting memory and the latter affecting cognitive functions other than memory. Individuals with aMCI have a higher rate of conversion to AD dementia (Grundman, 2004), and thus aMCI may represent preclinical stages of AD (Petersen et al., 2006). In contrast, individuals with naMCI have a higher rate of progression to other types of dementia, such as dementia with Lewy bodies (Ferman et al., 2013). Diagnostic assays to detect AD-related changes at MCI stage and differentiate aMCI from naMCI are therefore critical to identify patients at pre-clinical stage of AD for targeted early intervention. Significant progress has been made in the last few years in the clinical diagnosis of AD, such as the development of sensitive Single Molecular Array (Simoa) assays for site-specific phospho-tau epitope detection (such as p-tau181, p-tau217, and p-tau231) to differentiate AD from non-AD with brain tissues, CSF and blood samples (Rissin et al., 2010; Blennow et al., 2015; Janelidze et al., 2020; Ashton et al., 2021, 2022; Thijssen et al., 2021). However, MCI diagnosis and MCI subgroups discrimination remain a significant challenge.

In this study, we sought to evaluate the capabilities of Simoa p-tau181 test as a molecular biomarker for differential diagnosis of mild cognitive impairment of Alzheimer’s disease with matching plasma and CSF samples. Simoa p-tau181 test is a FDA-approved digitizing biomarker developed for low-abundance detection in plasma or CSF and translated for clinical diagnostic evaluation of Alzheimer’s disease (Mielke et al., 2018; Karikari et al., 2020). Current study focuses on p-tau181 utility for MCI diagnosis from cognitively normal cohorts and for MCI subgroups (aMCI and naMCI) discrimination, in part because p-tau181 is one of the best characterized molecular biomarker for AD diagnosis. Using a clinically diagnosed set of cognitively normal, aMCI, naMCI, and AD dementia cohorts (total 117 participants), we first demonstrated that p-tau181 is capable of differentiating MCI subjects from cognitively normal cohorts with high confidence in matching plasma and CSF samples (*p* = 0.0009 in plasma and *p* < 0.0001 in CSF). While our data showed that p-tau181 is able to diagnose aMCI, it is less capable to differentiate naMCI from cognitively normal individuals. Furthermore, our data demonstrated p-tau181 is not capable of differentiating aMCI from naMCI cohorts. Thirdly, either factor of being Aβ-positive or total tau-positive markedly improved differentiation performance to diagnose aMCI with p-tau181 from cognitively normal group.

## Materials and methods

### Study participants

Clinical diagnosis of dementia, MCI, and cognitively normal was based on a clinical evaluation at the Duke Memory Disorder clinic. Cognitively normal participants were recruited separately and their cognitive status was determined by the patients’ functional status. Cognitive status of MCI and AD patients was determined by a combination of patients’ (or their family members’) stated symptoms, their functional status (activities of daily living, ADLs, and instrumental activities of daily living, iADLs), and the Montreal Cognitive Assessment (MoCA) neuropsychological evaluation. Demographic information, clinical diagnosis, CSF Aβ and total tau biomarker data of total 117 participants included in this study are summarized in Table 1 and obtained from the Duke University Department of Neurology’s BioBank.

**TABLE 1:** Demographic and characteristics of study participants (*N* = 117).

	CN (35)	aMCI (34)	naMCI (17)	AD (31)	*P*-value
**Age** (mean ± SD), years	67.4 ± 16.8	72.1 ± 8.5	70.6 ± 9.7	72.7 ± 9.2	0.12 (CN vs. AD) 0.16 (CN vs. aMCI) 0.43 (CN vs. naMCI)
**Sex**, Female (%)	24 (68.6)	20 (58.8)	6 (35.3)	18 (58.1)	
**MoCA score** (mean ± SD)	NA	22.8 ± 2.3	22.2 ± 3.9	16.9 ± 5.1	9.7 × 10^–7^ (aMCI vs. AD) 3.1 × 10^–4^ (naMCI vs. AD)
**A**β**** (mean **±** SD), pg/ml	NA	1040.8 ± 420.1	1119.2 ± 495.6	815.9 ± 309.8	0.017 (aMCI vs. AD) 0.032 (naMCI vs. AD)
**Total tau** (mean ± SD), pg/ml	NA	282.9 ± 133.1	266.8 ± 175.2	309.8 ± 185.6	0.51 (aMCI vs. AD) 0.43 (naMCI vs. AD)

Abbreviations: CN, cognitively normal; aMCI, amnestic MCI; naMCI, non-amnestic MCI; AD, Alzheimer’s disease; MoCA, Montreal cognitive assessment test; NA, not available.

### Plasma and CSF sample collection and handling

Blood samples were collected and handled as described elsewhere (Palmqvist et al., 2019; Janelidze et al., 2020). Briefly, blood samples were collected at the same time as CSF samples, and the collection was performed during a clinically indicated lumbar puncture visit with participants not fasting. Blood samples were collected and analyzed according to a standardized protocol. For each study participant, blood was collected in EDTA-plasma tubes and centrifuged (2000 g, 4°C) for 10 min. After centrifugation, plasma from all tubes were combined, mixed, and 1 mL was aliquoted into polypropylene tubes and stored at −80°C within 30 to 60 min of collection. Lumbar CSF samples were collected through a standard spinal tap procedure performed at the Duke Memory Disorders clinic. The laboratory technicians performing the biochemical analyses of plasma and CSF biospecimens were blinded to the clinical data.

### Measurement of CSF Aβ and total tau levels

For the entire cohort, Aβ and total tau concentrations were measured using β-amyloid (1–42) and hTau Ag ELISA tests. Aβ42 and total tau levels were measured on the Roche Elecsys system. These measurements were performed by Clinical Laboratory Improvement Amendments (CLIA)-certified Mayo Clinic Laboratories following manufacturers’ instructions. CSF Aβ42 and total tau cut-offs follow Mayo Clinic reference values: Aβ42 at 1026 pg/ml and total tau cut-off at 238 pg/ml (Hansson et al., 2018).

### Matching plasma and CSF p-tau181 Simoa measurements

P-tau181 Simoa measurements were performed in a Quanterix HD-X instrumentation platform in the Molecular Genomics Core of the Duke Molecular Physiology Institute. Matching plasma and CSF p-tau181 measurements used high precision Quanterix Simoa p-tau181 Advantage V2 kit (cat #103714; Quanterix Corp., Billerica, MA, USA): 7.7% for coefficient of variation (CV) between runs, 6.5% for CV within runs, and 3.7% of CV between instruments. Calibrators were run in triplicates, internal low and high controls were measured in duplicates, and matching plasma and CSF p-tau181 Simoa were measured in singlicates. CSF samples were diluted 1:10 on bench manually using vendor’s diluent and all plasma samples used 1:4 dilution onboard. The coefficient of variation for an average number of enzyme per bead (AEB CV) for each calibrator was controlled within 20% per vendor’s instruction. *R*^2^ values for triplicated calibrator curve fitting were controlled with 0.95 or above.

### MoCA evaluation

The MoCA test is a brief cognitive screening tool with high sensitivity and specificity for detecting MCI as currently conceptualized in patients performing in the normal range on the mini-Mental State Examination (MMSE) (Nasreddine et al., 2005). It is a 10-min cognitive screening tool to assist first-line physicians in detection of MCI, a clinical state that often progresses to dementia.

### Statistical analysis

Plasma or CSF p-tau181 levels, or plasma/CSF p-tau181 ratios in cognitively normal, MCI, AD dementia or MCI subgroups with varying Aβ42 or total tau burden were plotted in box-whisker format in GraphPad Prism software (version 9.0). Each box-whisker plot shows levels for minimum (Q0), 25^th^ percentile (Q1), 50^th^ percentile (Q2 or median), 75^th^ percentile (Q3) and maximum (Q4). The differences between various comparing groups were analyzed with non-parametric Mann-Whitney test as implemented within GraphPad Prism software. Detailed statistical analysis results are listed in Supplementary Tables 1, 2. *P*-values < 0.05 were considered significant. The specificity and sensitivity of plasma or CSF p-tau181 with the specific comparing groups were determined based on the area under the curve (AUC) of receiver operating characteristics (ROC) analysis. The 95% confidence interval (CI_95%_) of AUC was calculated with Wilson/Brown method. Demographic factor of sex in relation to p-tau181 Simoa measurement levels in plasma or CSF was analyzed by non-parametric Mann-Whitney test in GraphPad Prism 9.3.1. *P*-values <0.05 were considered significant.

### Correlation analysis

The non-parametric Spearman’s correlation coefficients between p-tau181 levels versus age, p-tau181 levels in plasma versus CSF in cognitively normal (CN), MCI, or AD dementia groups, or CSF p-tau181 versus CSF Aβ or total tau level were calculated using GraphPad Prism 9.3.1. *R*-values > 0.60 are considered strong correlation, *r* values between 0.40–0.59 as moderate correlation, and *r* < 0.39 as weak or no correlation.

## Results

### Demographic and clinical characteristics

The basic demographic and clinical characteristics of the participants are shown in Table 1. The AD dementia group did not differ significantly in age from those for aMCI or naMCI groups. These groups were on average 3–5 years older than the cognitively normal group but these differences were not statistically significant (*p* = 0.12 for AD vs. CN, *p* = 0.16 for aMCI vs. CN, and *p* = 0.43 for naMCI vs. CN, respectively). The percentage of females in AD group (58.1%) is similar to that of aMCI group (58.8%). Cognitively normal group had higher female percentage (68.6%) and naMCI group had a lower percentage of females (35.3%). As expected, MoCA scores for the AD group were significantly lower than the aMCI or naMCI groups (*p* = 9.7 × 10^–7^ and *p* = 3.1 × 10^–4^, respectively); CSF Aβ42 levels were decreased in the AD group versus those in aMCI or naMCI groups (*p* = 0.017 or *p* = 0.032, respectively); and total tau levels were elevated but not statistically different in AD cohorts versus those in aMCI or naMCI groups (*p* = 0.51 and *p* = 0.43, respectively).

### P-tau181 is capable to diagnose both AD and MCI from cognitively normal subjects

**FIGURE 1:**
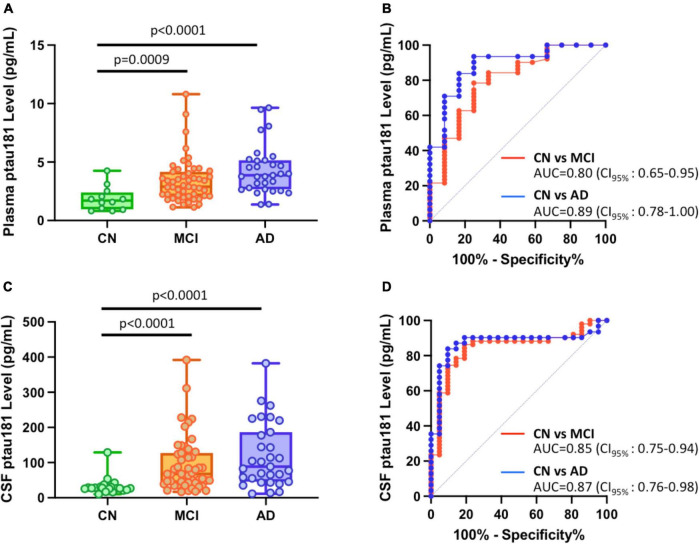
Differential capabilities of Simoa plasma and CSF p-tau181 measurements in cognitively normal (CN), MCI, and Alzheimer’s disease (AD) dementia cohorts. **(A,C)** Box and whisker plot of p-tau181 in plasma panel **(A)**, and CSF panel **(C)** for CN, MCI and AD subjects. **(B,D)** ROC curves discriminating among controls, MCI and AD dementia for the p-tau181 levels in plasma panel **(B)** and CSF panel **(D)**.

Significantly higher concentrations of both plasma and CSF p-tau181 were found in AD group (*n* = 31; *p* < 0.0001 for both plasma and CSF) and MCI cohort (*n* = 51; *p* = 0.0009 for plasma samples and *p* < 0.0001 for CSF samples) compared with cognitively normal group (*n* = 12 for plasma samples and *n* = 39 for CSF samples) (Figures 1A, C; Supplementary Table 1). Receiving operating characteristic (ROC) analyses for the classification of AD vs. CN or MCI vs. CN showed similarly significant differential power for plasma (AUC = 0.89 for AD vs. CN; AUC = 0.80 for MCI vs. CN), and CSF (AUC = 0.87 for AD vs. CN; AUC = 0.85 for MCI vs. CN) (Figures 1B, D).

### P-tau181 can robustly diagnose aMCI, but not naMCI, from cognitively normal subjects

**FIGURE 2:**
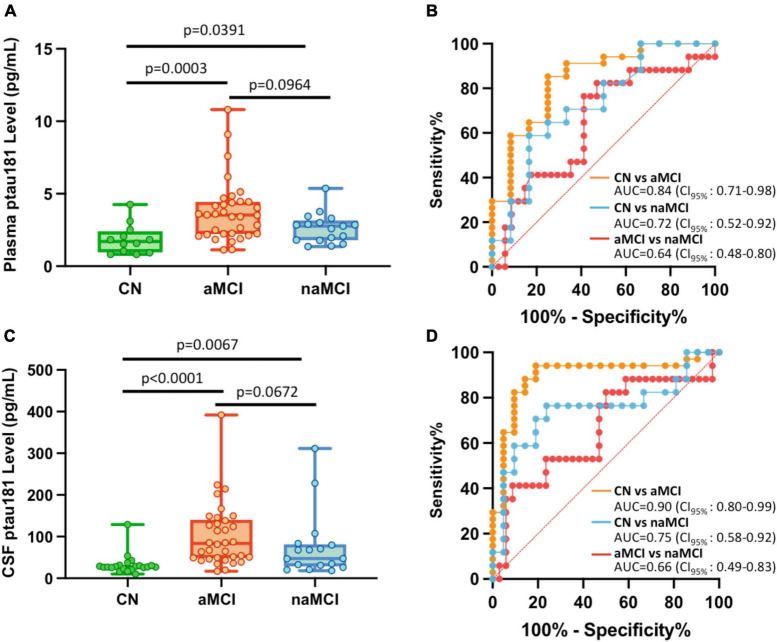
Differential capabilities of Simoa plasma and CSF p-tau181 measurements in cognitively normal (CN), amnestic MCI (aMCI), and non-amnestic MCI (naMCI) cohorts. **(A,C)** Box and whisker plot of p-tau181 in plasma panel **(A)**, and CSF panel **(C)** for CN, aMCI and naMCI subjects. **(B,D)** ROC curves discriminating among controls, aMCI and naMCI for the p-tau181 levels in plasma panel **(B)** and CSF panel **(D)**.

If MCI cohorts were broken down into aMCI and naMCI subgroups, differentiation between these two MCI subgroups are somewhat different from cognitively normal subjects. P-tau181 can robustly diagnose aMCI: its levels in aMCI were significantly higher than those in CN cohorts (*p* = 0.0003 for plasma samples and *p* < 0.0001 for CSF samples; Figures 2A, C; Supplementary Table 1) and ROC analyses for the classification of aMCI vs. CN showed high sensitivity and specificity (AUC = 0.84 for plasma samples and AUC = 0.90 for CSF samples; Figures 2B, D). However, differentiation of naMCI from CN cohorts was not as robust: even though p-tau181 levels were still significantly higher statistically (*p* = 0.0391 in plasma and *p* = 0.0067 in CSF; Figures 2A, C; Supplementary Table 1), separation in ROC analyses was only moderate (AUC = 0.72 in plasma and AUC = 0.75 in CSF; Figures 2B, D).

### P-tau181 is not capable to differentiate aMCI from naMCI cohorts

To determine if plasma or CSF p-tau181 levels can differentiate aMCI cohorts from naMCI subjects, p-tau181 levels of these two MCI subgroups were compared. We found that p-tau181 is not capable of differentiating aMCI from naMCI cohorts: p-tau181 levels were not statistically different between aMCI samples from naMCI samples (*p* = 0.0964 in plasma and *p* = 0.0672 in CSF; Figures 2A, C; Supplementary Table 1). Furthermore, ROC analyses demonstrated that p-tau181 levels, with its low discriminatory power in both plasma (AUC = 0.64; Figure 2B) and CSF samples (AUC = 0.66; Figure 2D), would not be clinically useful (AUC ≤ 0.75) to differentiate aMCI from naMCI cohorts.

### P-tau181 has high probability to diagnose Aβ-positive or total tau-positive aMCI subjects from cognitively normal subjects

**FIGURE 3:**
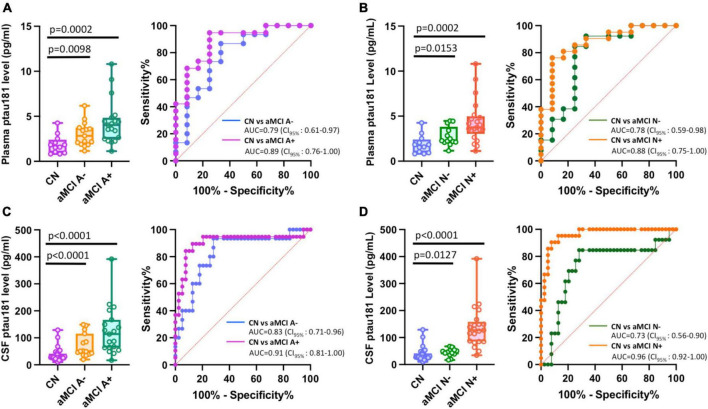
Impact of Aβ or total tau burden in differentiating among amnestic MCI (aMCI) subgroups using Simoa plasma or CSF p-tau181 measurements. **(A–D)** Left panels are box and whisker plots of p-tau181 in plasma panel **(A,B)**, and CSF panel **(C,D)** for cognitively normal groups versus aMCI subjects with positive or negative Aβ burdens (aMCI A+ or aMCI A−) or total tau burdens (aMCI N+ or aMCI N−). Right panels show ROC curves discriminating corresponding two aMCI subgroups for the p-tau181 levels in plasma or CSF.

We first investigated how Aβ or total tau burden each affects the differentiation of aMCI groups from cognitively normal cohort. While patients with Aβ-positive (aMCI A+) or Aβ-negative (aMCI A−), and total tau positive (aMCI N+) or negative (aMCI N−) groups all showed statistically higher plasma or CSF p-tau181 levels (Figures 3A–D, left panels; Supplementary Table 1), the differentiation capabilities of Aβ-negative or total tau negative groups vs. cognitively normal group remain relatively low (AUCs range from 0.73 to 0.83) compared with corresponding Aβ-positive and total tau positive groups (AUCs range from 0.88 to 0.96; Figures 3A–D, right panels). P-tau181 showed high probability to diagnose both Aβ-positive or total tau-positive aMCI groups from cognitively normal subjects (AUC ≥ 0.88).

**FIGURE 4:**
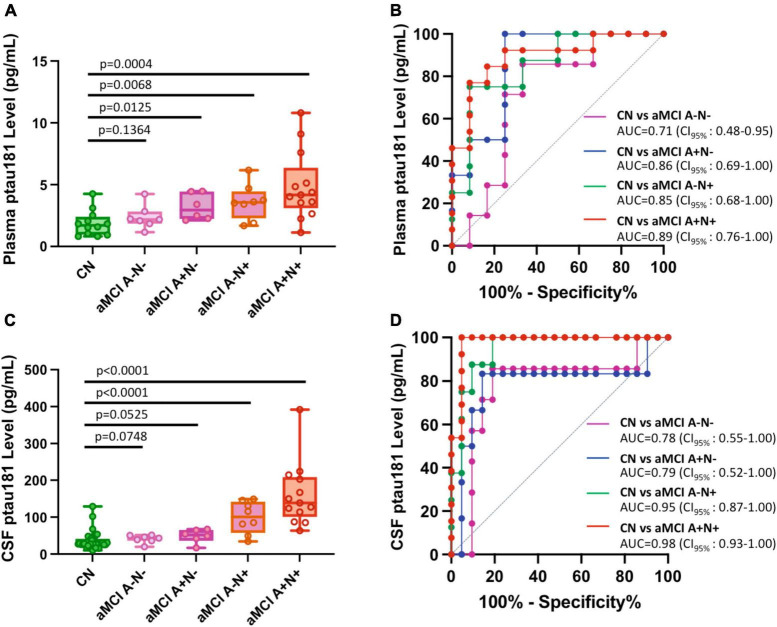
Differential capabilities of Simoa plasma or CSF p-tau181 levels in separating various amnestic MCI (aMCI) cohorts from cognitively normal (CN) subjects: Impact of Aβ and total tau burden. **(A,C)** Box and whisker plot of p-tau181 in plasma panel **(A)**, and CSF panel **(C)** for CN and aMCI subjects with positive or negative Aβ or total tau burdens. Panel **(B,D)** ROC curves discriminating among controls and various subclassified aMCI for the p-tau181 levels in plasma panel **(B)** and CSF panel **(D)**.

We further classified aMCI subgroups into two total tau-positive groups (aMCI A+N+ and aMCI A−N+) and two total tau-negative groups (aMCI A−N− and aMCI A+N−). We used “N+” or “N-” for total tau-positive or total tau-negative based on the ATN classification system (amyloid, tau, neurodegeneration) (Jack et al., 2018), where each individual is rated for the presence of β-amyloid (CSF Aβ or amyloid PET: “A”), hyperphosphorylated tau (CSF p-tau or tau PET: “T”), and neurodegeneration (CSF total tau or atrophy on structural MRI, fluorodeoxyglucose (FDG)-PET: “N”) (Ebenau et al., 2020). In this study, A, N status was determined based on CSF biomarker Aβ and total tau levels (see Materials and methods section). Our results showed that tau-positive aMCI subgroups have significantly higher p-tau181 concentrations in both plasma (*p* = 0.0004 for aMCI A+N+ vs. CN and *p* = 0.0068 for aMCI A−N+ vs. CN; Figure 4A; Supplementary Table 1) and CSF samples (*p* < 0.0001 for both aMCI A+N+ vs. CN and aMCI A−N+ vs. CN; Figure 4C; Supplementary Table 1). Corresponding ROC analyses also showed excellent discriminatory power for p-tau181: AUC = 0.89 for aMCI A+N+ vs. CN and AUC = 0.85 for aMCI A−N+ vs. CN in plasma measurements (Figure 4B); AUC = 0.98 for aMCI A+N+ vs. CN and AUC = 0.95 for aMCI A−N+ vs. CN in CSF measurements (Figure 4D). In contrast, tau-negative aMCI subgroups had less degree of significant elevation or did not show statistically significant elevation in p-tau181 concentrations in plasma (*p* = 0.0125 for aMCI A+N− vs. CN and *p* = 0.1364 for aMCI A−N− vs. CN; Figure 4A; Supplementary Table 1) and CSF samples (*p* = 0.0525 for aMCI A+N− vs. CN and *p* = 0.0748 for aMCI A−N− vs. CN; Figure 4C; Supplementary Table 1). Corresponding ROC analyses also showed less prominent discriminatory power for p-tau181: AUC = 0.86 for aMCI A+N− vs. CN and AUC = 0.71 for aMCI A−N− vs. CN in plasma measurements (Figure 4B); AUC = 0.79 for aMCI A+N− vs. CN and AUC = 0.78 for aMCI A−N− vs. CN in CSF measurements (Figure 4D). Due to relatively small number of total naMCI cohort (n = 17), subgroup classifications of naMCI group into Aβ and total tau biomarker positive or negative was not performed.

### Plasma/CSF p-tau181 ratio as a potential biomarker for differentiation

We further evaluated the ratio of plasma/CSF of p-tau181 for matching biofluid samples to test if such ratio could be a useful biomarker for MCI differentiation. We found while the aMCI cohort shows a trend of reduced plasma/CSF ratio compared to those of cognitively normal cohort or naMCI cohort, such reduction was not enough to be statistically significant (*p* = 0.069 for CN vs. aMCI; *p* = 0.215 for aMCI vs. naMCI; Supplementary Figure 1A; Supplementary Table 2). In separate comparisons of aMCI vs. CN (*p* = 0.106), aMCI A+ vs. CN (*p* = 0.0268), and aMCI N+ vs. CN (*p* = 0.0035), we found Aβ and total tau positivity improve the differentiation power of p-tau181 to separate aMCI from cognitively normal cohort (Supplementary Figure 1B; Supplementary Table 2). This data further supports our existing conclusion above that p-tau181 has high probability to diagnose Aβ-positive or total tau-positive aMCI subjects from cognitively normal subjects (Figure 3).

### CSF p-tau181 has strong, positive correlation with total tau burden in both aMCI and naMCI cohorts but weak and negative correlation with Aβ burden in both MCI subgroups

**FIGURE 5:**
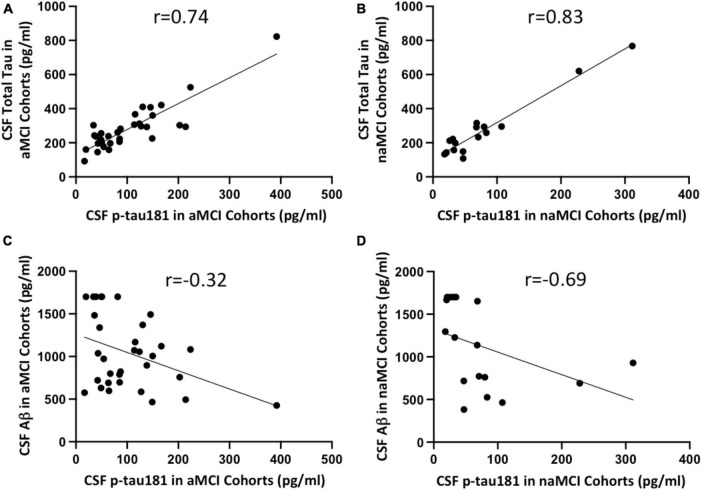
Correlation between CSF Simoa p-tau181 and CSF total tau in aMCI **(A)** and naMCI **(B)** cohorts, and between CSF Simoa p-tau181 and CSF Aβ in aMCI **(C)** and naMCI **(D)** cohorts. Correlation coefficient *r* values were calculated using non-parametric Spearman’s correlation tests.

We investigated if CSF p-tau181 level in aMCI or naMCI cohorts correlated with CSF total tau or Aβ burden, or other demographic factors such as sex and age. Not surprisingly, our data showed CSF p-tau181 levels have strong and positive correlation with CSF total tau burden in both aMCI (*r* = 0.74; Figure 5A) and naMCI cohort (*r* = 0.83; Figure 5B). Interestingly, CSF p-tau181 levels were negatively correlated with CSF Aβ burden in aMCI cohort (*r* = −0.32; Figure 5C) or in naMCI cohort (*r* = −0.69; Figure 5D). Plasma and CSF p-tau181 levels have no significant differences between male versus female in both aMCI and naMCI cohorts (*P*-values range from 0.29 to 0.77; Supplementary Figure 2). In terms of demographic factor of age, plasma and CSF p-tau181 levels have no correlations with age in aMCI cohort (*r* = 0.21 or *r* = 0.20; Supplementary Figures 3A, C). Plasma and CSF p-tau181 levels have moderate correlation with naMCI group (*r* = 0.55 or r = 0.48; Supplementary Figures 3B, D). Due to limited data of MoCA scores (not every patient completed the MoCA test or was available to review), correlation between p-tau181 with MoCA score was not evaluated.

### Plasma and CSF p-tau181 levels have significant correlation with each other

We further investigated if CSF p-tau181 levels correlated with plasma p-tau181 concentrations in various subgroups or combined groups. Our data suggested CSF p-tau181 concentration has significant, positive correlation with corresponding cohort’s plasma p-tau181 levels: *r* = 0.76 for CN group (Supplementary Figure 4A); *r* = 0.56 for MCI group and *r* = 0.55 for aMCI group (Supplementary Figures 4B, D); *r* = 0.49 for AD dementia group (Supplementary Figure 4C); and *r* = 0.63 for combined CN, MCI, and AD groups (Supplementary Figure 4E).

## Discussion

Recently, there is a growing research interest in early diagnosis and intervention for individuals with mild cognitive impairment (MCI). Diagnostic assays to detect AD-related changes at MCI stage are critical to identify patients at preclinical stage of AD for targeted early intervention. Furthermore, new biomarkers to differentiate subclasses of MCI (aMCI and naMCI), which will likely develop into different types of dementias, are of high translational utilities for targeted therapy in clinical healthcare. While much remains to be investigated, several recent p-tau biomarker studies began to test their potential for MCI diagnosis and progression from MCI to AD dementia. Plasma p-tau181 levels were found to be elevated in Aβ+ cognitively unimpaired, Aβ+ MCI, and Aβ+ AD dementia compared to those in Aβ− cognitively unimpaired and non-AD disease groups (Janelidze et al., 2020). In a separate study, plasma p-tau181 and PET centiloid scale alone or in combination with other biomarkers were found to produce high predictive value in predicting future cognitive stage transition (Kwon et al., 2023). CSF p-tau235 levels were elevated in amyloid-positive MCI cases compared to amyloid-negative cognitively unimpaired cohorts (Lantero-Rodriguez et al., 2021). A recent study of p-tau217 study comparing 10 plasma p-tau assays in prodromal AD cohorts suggested that mass-spectrometry-based measures of p-tau217 was able to identify MCI patients who will subsequently progress to AD dementia with high probability (AUC = 0.932) whereas other non-mass spectrometry-based Simoa, Lumipulse immunoassay, Meso Scale Discovery immunoassay, and Splex immunoassay yielded wide AUC range from 0.688–0.889 in predicting future progression to AD dementia (Janelidze et al., 2023). A recent study suggested plasma p-tau217 predicted cognitive decline in patients with pre-clinical AD (Mattsson-Carlgren et al., 2023). To our knowledge, no p-tau biomarker studies have been reported on diagnosing MCI subclasses aMCI and naMCI from cognitively normal subjects, and on discriminating aMCI from naMCI cohorts.

Our data suggest that p-tau181 is not sensitive enough to differentiate naMCI from cognitively normal cohort and to discriminate aMCI from naMCI as in both cases the discriminating power scores were well below 0.80 (AUC = 0.72 in plasma samples and AUC = 0.75 in CSF samples for naMCI vs. cognitive normal group; AUC = 0.68 in plasma samples and AUC = 0.66 in CSF samples for aMCI vs. naMCI). It is unclear if the relatively small case number of naMCI (*n* = 17) affected the discriminating power. Therefore, future studies with larger cohort number of naMCI should be evaluated to further clarify this question. More importantly, other p-tau biomarkers should be evaluated or new p-tau biomarkers should be developed for these differential translational applications. Several p-tau biomarkers (p-tau217, p-tau231, and p-tau235) are well-established markers to diagnose AD dementia from non-AD controls. A recent meta-analysis suggested that p-tau217 had better discriminative accuracy for MCI than p-tau181 and p-tau231 (Chen et al., 2022). We recently showed that a new neuropathological biomarker p-tau198 had capability to diagnose MCI cohorts from cognitively normal subjects in brain tissues (Wu et al., 2022a). Therefore, it will be interesting in the future to evaluate these promising p-tau biomarkers or to develop novel p-tau biomarkers for plasma and CSF tests.

Our analysis demonstrated that aMCI diagnosis with p-tau181 biomarker noticeably improves in the Aβ-positive (aMCI A+ and aMCI N+ in Figure 3), total tau-positive MCI cohorts (aMCI A−N+ and aMCI A+N+ in Figure 4), or using plasma/CSF p-tau181 ratio as a biomarker (Supplementary Figure 1B). This is consistent with common clinical practice of using a combination of biomarkers (site-specific p-tau, Aβ, total tau, ApoE ε4 etc.) for more accurate diagnosis. Multi-factorial nature of AD disease etiology has been well recognized with respect to AD biomarker and therapeutic development (Gustaw-Rothenberg et al., 2010; DeTure and Dickson, 2019). It is intriguing that Aβ-positive and total tau-negative aMCI group has nearly the same differentiation power as Aβ-negative and total tau-negative aMCI from cognitively normal group (AUC = 0.79 for the former and 0.78 for the latter, Figure 4D) whereas Aβ-positive aMCI group has noticeably higher differentiation power than Aβ-negative aMCI group from cognitively normal group (AUC = 0.91 for the former and 0.83 for the latter, right panel of Figure 3C). We speculate the cause for this difference is that our Aβ-positive aMCI group (aMCI A+ in Figure 3) contains both total tau negative or total tau positive cohorts, and highly significant elevation in p-tau181 levels in Aβ− and total tau-double positive aMCI cases vs. cognitively normal group compensated for the very little elevation in p-tau181 levels in Aβ-positive and total tau-negative aMCI cohorts vs. CN cohort (Figures 4C, D).

Over fifty post-translational modification (PTM) sites in tau protein have been identified, which include dozens of phosphorylation sites, and multiple sites with other modifications such as acetylation and ubiquitination (Wesseling et al., 2020). PTMs of tau proteins lead to structural and molecular diversity that may be linked to disease staging and contribute to AD clinical heterogeneity and disease progression (Arakhamia et al., 2020; Dujardin et al., 2020; Xia et al., 2021). Different tau PTMs may occur at different AD stages in a sequential fashion, and specific combinations of PTMs were proposed to reflect progressive steps in the process of tau fibril formation and AD disease progression (Wesseling et al., 2020; Wu et al., 2022a). Therefore, more sensitive and specific p-tau or other tau PTM-based biomarkers may remain to be discovered. Alternatively, other sensitive detection methods applicable to amplify misfolded protein seeds, such as real-time quaking-induced conversion (RT-QuIC) and protein misfolding cyclic amplification (PMCA) may be developed and applied to the challenging MCI diagnosis with accessible plasma and CSF biospecimens (Salvadores et al., 2014; Wang et al., 2019, 2023; Wu et al., 2022b).

## Conclusion

We showed that plasma and CSF p-tau181 levels can robustly differentiate MCI or aMCI from cognitively normal cohorts, but such differentiation is weaker in diagnosing naMCI from cognitively normal groups. Furthermore, p-tau181 biomarker is not capable of differentiating aMCI from naMCI cohorts. Thirdly, incorporation of either factor of Aβ or total tau burden markedly improved differentiation power to diagnose aMCI from cognitively normal group. Therefore, we conclude that plasma and CSF p-tau181 levels are a promising biomarker for diagnosing aMCI from normal controls in the preclinical phase. But more robust new biomarkers are needed to differentiate naMCI from cognitively normal cases or to discriminate between MCI subtypes, aMCI from naMCI.

## Data availability statement

The original contributions presented in the study are included in the article/Supplementary material, further inquiries can be directed to the corresponding authors.

## Ethics statement

The studies involving humans were approved by the Duke University IRB; North Carolina Central University IRB. The studies were conducted in accordance with the local legislation and institutional requirements. All participants of the Duke Department of Neurology Biospecimen Bank provided written informed consent. All human samples obtained from the Biospecimen Bank were de-identified and without any protected health information.

## Author contributions

LW: Data curation, Formal analysis, Funding acquisition, Investigation, Methodology, Validation, Visualization, Writing−original draft, Writing−review and editing. SA: Data curation, Investigation, Methodology, Validation, Writing−review and editing. S-HJW: Data curation, Formal analysis, Funding acquisition, Investigation, Methodology, Validation, Writing−review and editing. AL: Data curation, Formal analysis, Investigation, Methodology, Project administration, Resources, Supervision, Validation, Writing−review and editing. BX: Conceptualization, Data curation, Formal analysis, Funding acquisition, Investigation, Methodology, Project administration, Resources, Supervision, Validation, Visualization, Writing−original draft, Writing−review and editing.

## Supplementary material

Supplementary Figure 1 Differential capabilities based on plasma/CSF p-tau181 ratio. **(A)** Box and whisker plot of percent ratio of plasma/CSF p-tau181 in cognitively normal, aMCI, and naMCI cohorts. **(B)** Box and whisker plot of percent ratio of plasma/CSF p-tau181 in cognitively normal, aMCI, aMCI A+ and aMCI N+ cohorts. Statistical *P*-values in both panels were calculated using non-parametric Mann-Whitney test.

Supplementary Figure 2 Scatter plots of the p-tau181 levels in aMCI or naMCI cohorts in relation with demographic factor sex. Upper panels **(A,B)** show Simoa p-tau181 measurements in plasma and lower panels **(C,D)** show corresponding p-tau181 levels in CSF. Statistical *P*-values were calculated using non-parametric Mann-Whitney test.

Supplementary Figure 3 Scatter plots of the p-tau181 levels in aMCI or naMCI cohorts in relation with demographic factor age. Upper panels **(A,B)** show Simoa p-tau181 measurements in plasma and lower panels **(C,D)** show corresponding p-tau181 levels in CSF. Correlation coefficient *R* values were calculated using non-parametric Spearman’s correlation tests.

Supplementary Figure 4 Correlation between plasma and CSF Simoa p-tau181 measurements in the individual cohort of **(A)** cognitively normal (CN), **(B)** MCI, **(C)** AD dementia, **(D)** amnestic MCI (aMCI), and **(E)** combined cohorts of CN, MCI, AD subjects. Correlation coefficient *r* values were calculated using non-parametric Spearman’s correlation tests.
