# Supplementary material for: Differential diagnosis of mild cognitive impairment of Alzheimer’s disease by Simoa p-tau181 measurements with matching plasma and CSF
Source: Front Mol Neurosci. 2024 Jan 8;16:1288930. doi: 10.3389/fnmol.2023.1288930 (PMC10800554; doi:10.3389/fnmol.2023.1288930)
Supplement: Supplementary Figure 1 — Differential capabilities based on plasma/CSF p-tau181 ratio. (A) Box and whisker plot of percent ratio of plasma/CSF p-tau181 in cognitively normal, aMCI, and naMCI cohorts. (B) Box and whisker plot of percent ratio of plasma/CSF p-tau181 in cognitively normal, aMCI, aMCI A+ and aMCI N+ cohorts. Statistical P-values in both panels were calculated using non-parametric Mann-Whitney test. [file Data_Sheet_1.docx]

Supplemental Information

**Differential Diagnosis of Mild Cognitive Impairment of Alzheimer’s Disease by Simoa p-tau181 Measurements with Matching Plasma and CSF**

Ling Wu^1,5^, Stephanie Arvai^3^, Shih-Hsiu J. Wang^3,4,5^, Andy J. Liu^3,4,5,^*, and Bin Xu^1,2,5,^*

^1^Biomanufacturing Research Institute and Technology Enterprise (BRITE), North Carolina Central University, Durham, NC 27707, USA, ^2^Department of Pharmaceutical Sciences, North Carolina Central University, Durham, NC 27707, USA, ^3^Department of Neurology, ^4^Department of Pathology, Duke University Medical Center, Durham, NC 27705, USA, ^5^Affiliated Member, Duke/UNC Alzheimer’s Disease Research Center, Box 3003, Durham, NC 27710, USA

Supplemental Information contains two supplemental tables and four supplemental figures.

Abbreviations: CN, cognitively normal; MCI, mild cognitive impairment; aMCI, amnestic MCI; naMCI, non-amnestic MCI; AD, Alzheimer’s disease; CSF, cerebrospinal fluid.

Abbreviations: CN, cognitively normal; aMCI, amnestic mild cognitive impairment; naMCI, non-amnestic MCI; aMCI A+: Aβ-positive aMCI; aMCI N+: total tau positive aMCI; CSF, cerebro-spinal fluid.

**
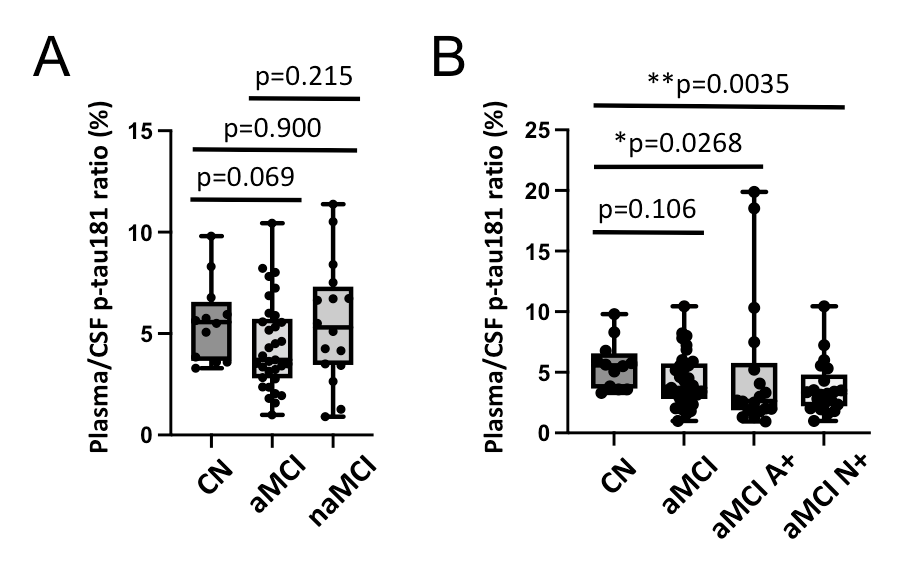
**

**Figure S1.** Differential capabilities based on plasma/CSF p-tau181 ratio. (A) Box and whisker plot of percent ratio of plasma/CSF p-tau181 in cognitively normal, aMCI, and naMCI cohorts. (B) Box and whisker plot of percent ratio of plasma/CSF p-tau181 in cognitively normal, aMCI, aMCI A+ and aMCI N+ cohorts. Statistical p values in both panels were calculated using nonparametric Mann-Whitney test.

**
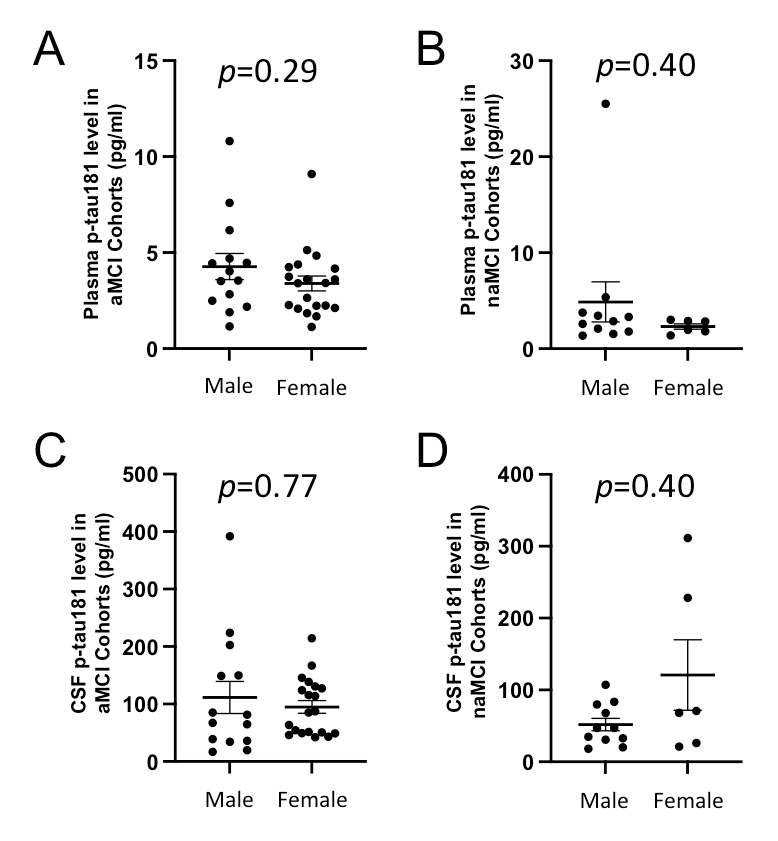
**

**Figure S2.** Scatter plots of the p-tau181 levels in aMCI or naMCI cohorts in relation with demographic factor sex. Upper panels (A,B) show Simoa p-tau181 measurements in plasma and lower panels (C,D) show corresponding p-tau181 levels in CSF. Statistical p values were calculated using nonparametric Mann-Whitney test.

**
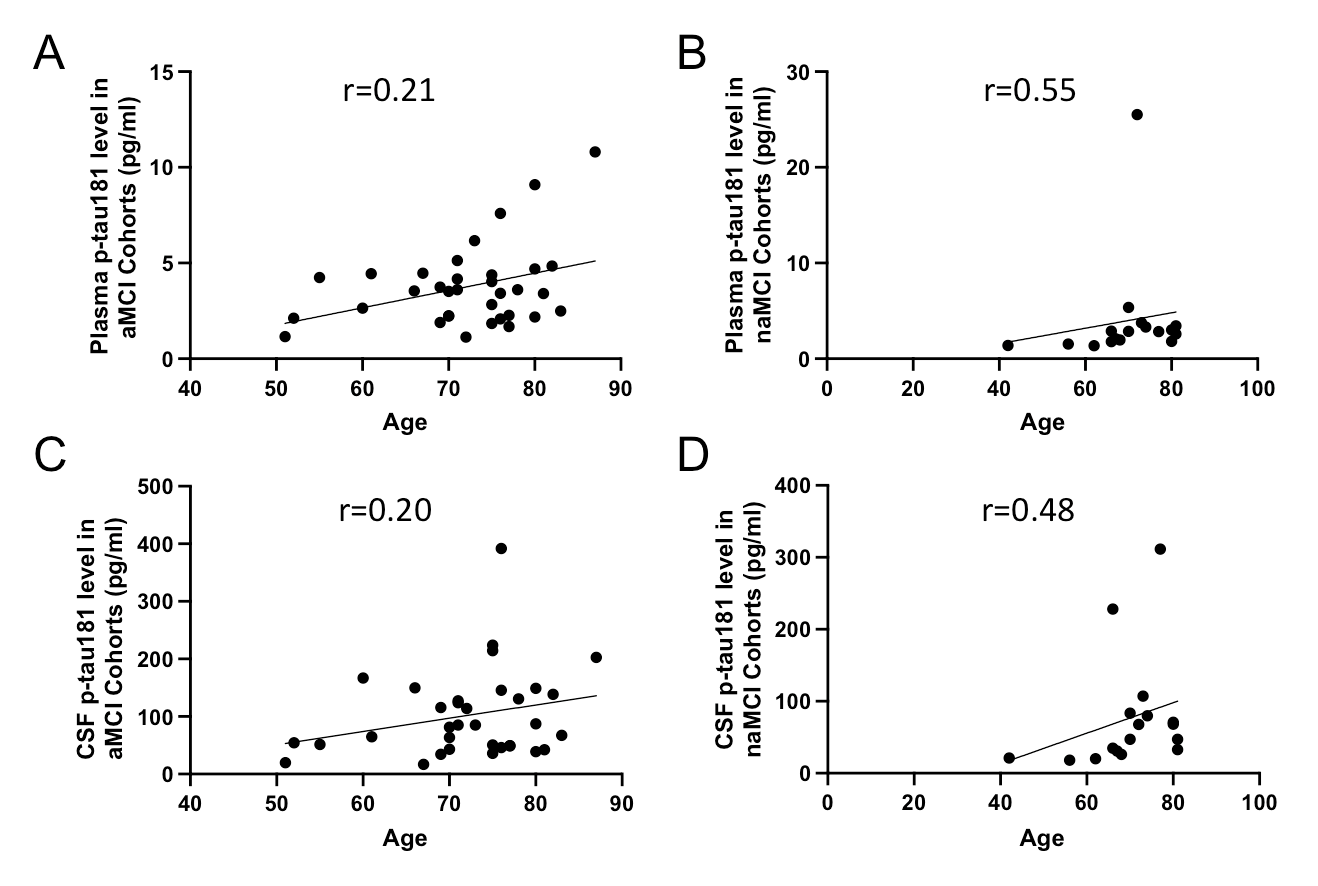
**

**Figure S3.** Scatter plots of the p-tau181 levels in aMCI or naMCI cohorts in relation with demographic factor age. Upper panels (A,B) show Simoa p-tau181 measurements in plasma and lower panels (C,D) show corresponding p-tau181 levels in CSF. Correlation coefficient r values were calculated using nonparametric Spearman’s correlation tests.

**
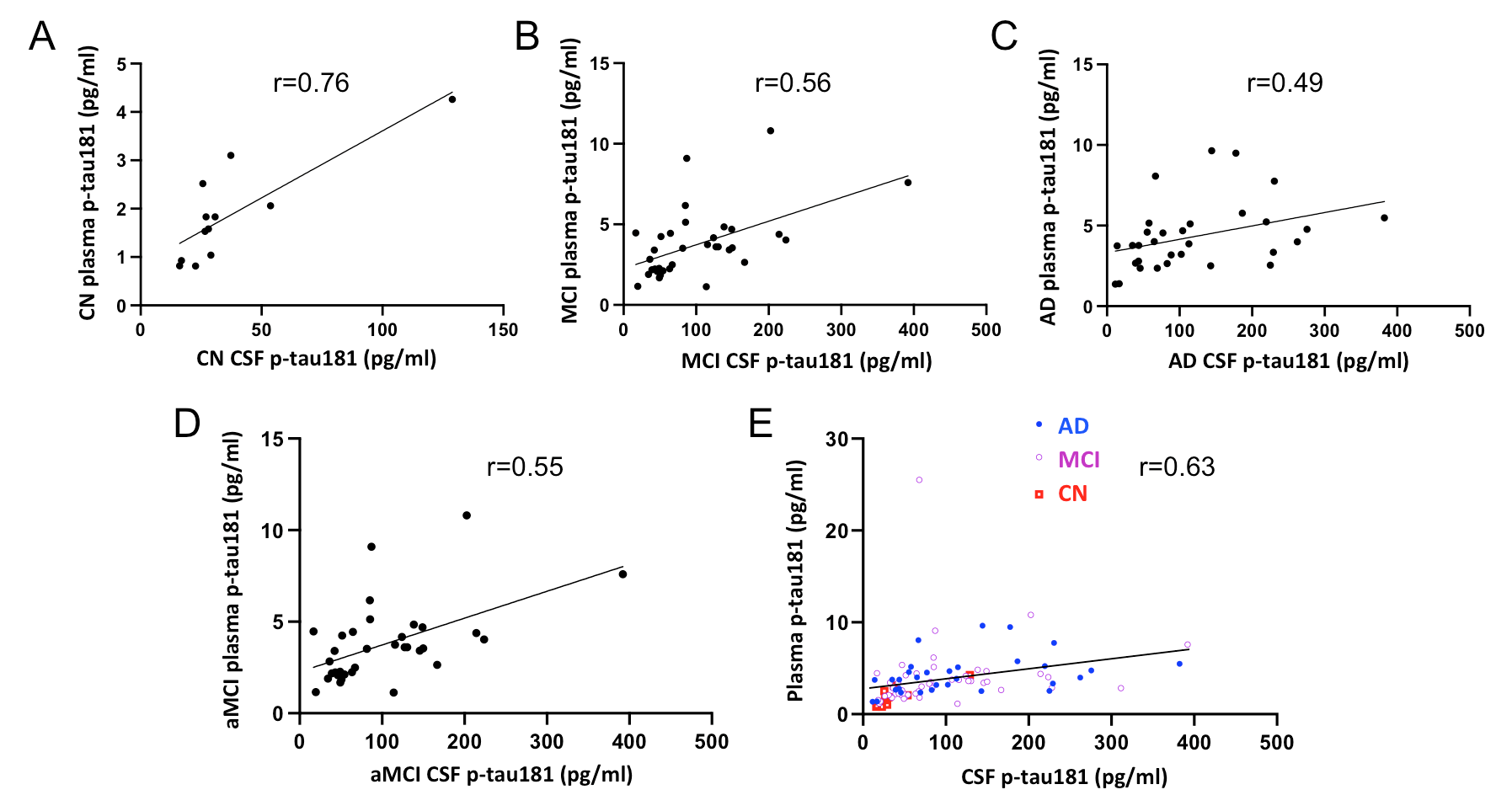
**

**Figure S4.** Correlation between plasma and CSF Simoa p-tau181 measurements in the individual cohort of (A) cognitively normal (CN), (B) MCI, (C) AD dementia, (D) amnestic MCI (aMCI), and (E) combined cohorts of CN, MCI, AD subjects. Correlation coefficient r values were calculated using nonparametric Spearman’s correlation tests.
